# Close of Session and Graduating Exercises

**Published:** 1855-03

**Authors:** 


### CLOSE OF SESSION AND GRADUATING EXERCISES.

The usual Lecture session of the Iowa Medical Department closes upon the last of February, just as this form of our Journal goes to press. The session has continued during the full term of four months, and has been faithfully attended by a studious and courteous class. It may not be improper to mention that three years ago, under a different organization from the present, the whole number of students attending lectures was fifteen, and the number of graduates six. During the session of last year, the number of matriculants was 41, while the whole number in actual attendance was about 50; at that session, the number of graduates was thirteen. But, during the session just closed, the class has amounted to about 75, and 19 graduates have just passed forth from our halls. This brief recapitulation is full of encouragement to us, and we are assured from the tenor of frequent letters that the healthy condition of the Institution thus indicated, besides being eminently satisfactory to us, affords pleasure to our very numerous friends in every portion of the State.

The closing exercises of the late session were held in this city on Tuesday evening, the 26th of February. After appropriate preliminary exercises, the charge to the graduates was pronounced by Dr. M'Gugin, who then bestowed Diplomas upon the following young gentlemen:

GRADUATING CLASS:

| Names. | Residence. | Subject of Thesis. |
|---|---|---|
| CHARLES R. ARNOLD, | *Conn.*, | The Blood. |
| ENOS T. BONNEY, | *Mo.*, | Colo-Rectitis. |
| JOHN R. CHAMBERS, | *Ill.*, | Chemical Diuretics. |
| D. S. COOK, | *Iowa*, | Dropsical Diseases. |
| WILLIAM T. DAY, | " | Protochloride of Mercury. |
| FRANCIS A. DORR, | " | Mechanism of Parturition. |
| JOHN J. ELLISON, | " | Uterine Hemorrhage. |
| T. H. EVERTS, | *Ind.*, | Relations of Life. |
| HENRY C. GROVER, | " | Physiology of Death. |
| J. W. HENDERSON, | *Ill.*, | Typhoid Fever. |
| JOHN A. HOLTON, | " | One Idea Systems of Med. |
| WM. F. HARLE, | *Iowa*, | Typhoid Fever. |
| JEFFERSON G. HART, | *Ky.*, | Koino-Miasmata. |
| J. M. MCKESSON, | *Ill.*, | Physiology of Respiration. |
| ROBT. PUTNAM, | *Iowa*, | Miasmata: Causes and Effects. |
| D. H. ROUSSEAU, | " | Typhoid Fever. |
| J. R. STANDLEY, | " | General Anatomy. |
| WM. N. TOWNDROW, | " | Gonorrhœa. |
| CALVIN C. WILKIE, | " | Pneumonia. |

The Honorary Degree of M. D., was also conferred upon the following gentlemen: Drs. N. UDELL, AMOS WITTER, C. C. GREEN, of Iowa, and Dr. J. C. PATTERSON, of Ill. The *ad eundem* degree, was conferred on Dr. GEO. W. TAYLOR, of Mo.

After the ceremony of conferring the degrees, followed the address of Prof. Sanborn, which was a very able production. The burthen of his theme was the necessity of self-reliance on the part of the physician, and his subject was not only appropriate and well chosen, but his position most ably sustained. This element of success and true greatness was most forcibly exhibited, and those who could look around them and back upon the pages of history, particularly of our own country, would find abundant evidence of the truth of the position taken by the speaker, viz., that it was the most efficient and active agent in eliminating and bringing out the brightest intellects and most useful and efficient minds of the past or the present day.

He also urged upon the consideration of the class, the necessity of the cultivation of the most incorruptible morals. He dwelt in particular upon honesty as a virtue which should be most sedulously observed and practiced. He alluded to the numerous opportunities of the physician to practice charlatanism, and referred to the lamentable want of knowledge in the secular world, relating to medicine, and that in this more than in any other pursuit, were the masses more liable to be deceived or imposed upon. In a most impressive manner he warned them against such low and disgraceful practices, but which to the dishonor of the profession are but too frequently adopted.

We can hardly say too much in praise of this effort either with regard to the matter or the manner. It has added another to the numerous evidences heretofore exhibited, of the talents and acquirements of the speaker; and in conclusion, we would say to our readers, that we shall hereafter cull out certain portions for our pages in the future, that they may enjoy a share of the intellectual treat with which those present were regaled.

After Prof. Sanborn, followed Prof. Hughes, who made a short statement of the history of the School, since its connection with the Iowa University, and the number of classes of each winter up to the present time. He also called the attention of the audience to the amount of labor performed by the Professors, and showed the number of lectures delivered in the last four months, to have been over six hundred, or equal to one hundred to each chair, besides enumerating other facts connected with our course next session.

Medical College Museum.—A very valuable contribution has recently been made to the Museum of the Medical Department, by Prof. Hughes. Messrs. A. H. & E. W. Winans, of Hamilton, Ill., opposite Keokuk, have, for some years, been devoted to the beautiful art of Taxidermy, and have, by long continued labor, prepared a great number of excellent specimens of Natural History, chiefly illustrative of ornithology. Dr. Hughes visited their collection a few weeks since, and was so much pleased with the beauty of the preparations, and so anxious that it should become a part of the College Museum, that he purchased the whole number, and has had them removed to the College buildings, where they are to be properly arranged in suitable cases, and set forth in all the beauty and grace of life-like expression. Among these are many rare and intrinsically valuable specimens, which deserve public preservation. We may mention the Otter, a magnificent Swan, Great Northern Diver, Snowy Owl, Great Horned Owl, Wild Turkey, Wild Cat, a beautiful young Deer, an Arboreal Coluber, Diamond Rattle Snake, and a Massasauga, with about a hundred other specimens. We are much pleased that so valuable a collection has been secured for our Museum, and are confident that another such cabinet does not exist in the State, and probably not in the whole north-western portion of the Union. The excellence of the preparation reflects much credit upon the almost self-taught artists, who have bestowed upon these specimens so much time and attention.

We have mentioned before, and take the liberty of repeating it, that we should be exceedingly thankful to any of our medical or scientific friends in the neighboring country, who may find it convenient to favor us with interesting specimens of Natural History or Geology, and particularly any that may illustrate Medical science, in any of its numerous branches.

We have received some donations, for which we are very thankful, and hope to be equally or more grateful for many others yet to follow.

---

We have received a number of recent publications, but too late for notice in the present number; among them the following:

"Report of the Sanitary Commission of New Orleans, on the Epidemic Yellow Fever of 1853"—from Dr. E. H. Barton. "Autobiography of Chas. Caldwell, M. D."—from Lippincott, Grambo & Co., Philadelphia. "Pathology and Treatment of Pulmonary Tuberculosis by John Hughes Bennett, M. D."—from Blanchard & Lea, Philadelphia. "Transactions of the New Hampshire Medical Society," and some other works, which we will notice in our next.

